# Population collapse of a Gondwanan conifer follows the loss of Indigenous fire regimes in a northern Australian savanna

**DOI:** 10.1038/s41598-022-12946-3

**Published:** 2022-05-31

**Authors:** David M. J. S. Bowman, Grant J. Williamson, Fay H. Johnston, Clarence J. W. Bowman, Brett P. Murphy, Christopher I. Roos, Clay Trauernicht, Joshua Rostron, Lynda D. Prior

**Affiliations:** 1 School of Natural Sciences, University of Tasmania, Sandy Bay, Private Bag 55, Hobart, TAS 7001 Australia; 2 Menzies Institute for Medical Research, University of Tasmania, Hobart, TAS 7000 Australia; 3 Research Institute for the Environment and Livelihoods, Charles Darwin University, Darwin, NT 0909 Australia; 4 Department of Anthropology, Southern Methodist University, Dallas, TX 75275 USA; 5 Department of Natural Resources and Environmental Management, University of Hawaii at Manoa, Honolulu, HI 96822 USA; 6 Korlorbirrahda Outstation, Maningrida, NT 0822 Australia

**Keywords:** Fire ecology, Sustainability, Ecology, Ecology, Environmental sciences, Environmental social sciences

## Abstract

Colonialism has disrupted Indigenous socioecological systems around the globe, including those supported by intentional landscape burning. Because most disruptions happened centuries ago, our understanding of Indigenous fire management is largely inferential and open to debate. Here, we investigate the ecological consequences of the loss of traditional Aboriginal fire management on fire-exposed savannas on the Arnhem Plateau, northern Australia, using the fire-sensitive conifer *Callitris intratropica* as a bio-indicator. We contrast Kakadu National Park, where traditional Aboriginal fire management was severely disrupted during the early twentieth century following Aboriginal relocation to surrounding settlements, and an adjacent Aboriginal estate where traditional Aboriginal fire management endures. Since 2006, traditional Aboriginal fire management at this site has been overlaid by a program of broad-scale institutionalized burning in the early dry season, designed to reduce greenhouse emissions. Using remote sensing, field survey, and dendrochronology, we show that on the Aboriginal estate, *C. intratropica* populations depend on the creation of a shifting patch mosaic of long unburned areas necessary for the recruitment of *C. intratropica.* However, the imposition of broad-scale fire management is disrupting this population patch dynamic. In Kakadu, there have been extreme declines of *C. intratropica* associated with widespread fires since the mid twentieth century and consequent proliferation of grass fuels. Fire management in Kakadu since 2007, designed to increase the size and abundance of patches of unburned vegetation, has not been able to reverse the population collapse of *C. intratropica*. Our study demonstrates that colonial processes including relocation of Indigenous people and institutional fire management can have deleterious consequences that are nearly irreversible because of hysteresis in *C. intratropica* population dynamics.

## Introduction

The current surge of uncontrolled and economically disastrous wildfires around the world calls into question how contemporary societies can sustainably co-exist with landscape fires^1,2^. It is well understood that inappropriate fuel management and poor landscape planning, combined with climate change, are major contributors amplifying the natural hazard of fires in flammable landscapes^2,3^. Uncontrolled fires have also been attributed to the collapse of Indigenous fire management practices, leading to discussion as to whether ‘cultural burning’ systems, based on traditional Aboriginal fire management practices, can be applied to mitigate wildfires driven by climate change^4^. For instance, the Australian Government inquiry into the catastrophic Australian fires of 2019‒2020 highlighted the potential importance of cultural burning in building natural disaster resilience and improving the effectiveness of the management of public lands^5^. What is overlooked in discussions is that understanding of the ecological outcomes of Indigenous fire management regimes is scientifically incomplete, geographically patchy, and often based on the interpretation of ambiguous historical accounts and analysis of palaeoecological archives such as tree rings and lake sediments. Inferences based on these historical proxies are debatable and sometimes controversial^6^. Importantly, the design and implementation of sustainable fire regimes is hampered because palaeoecological time-series are typically unable to illuminate the fine-scale spatial pattern of fires^7^.

The drastic socio-ecological disruptions that followed colonization and conquest since the 1500s^8^ mean that globally there are very few flammable landscapes remaining with an unbroken tradition of Indigenous fire management. Colonization processes not only include direct violence, Indigenous population collapse, relocation, dispossession, forced acculturation, and political and economic subjugation, but also the imposition of the worldviews of colonial powers and institutions that represent those worldviews, such as National Parks, formal wilderness areas, and institutional fire management programs that have typically excluded Indigenous people^9^. Many modern wildfire problems are a product of some combination of these complex and historically-contingent colonial processes. These processes are ongoing, and the ecological effects of colonialism continue to evolve even in contexts with unbroken histories of Indigenous fire management. These disruptions mean there have been remarkably few detailed observational and ecological studies of Indigenous fire regimes globally.

Australia, with its flammable biota, was colonized relatively late in the European colonial endeavor, so it necessarily holds a very prominent place in understanding Indigenous fire practices^6^. The colonialization of Australia commenced in 1788 and settler-expansion reached the Northern Territory nearly a century later. Throughout Australia colonialism has had catastrophic impacts on most Indigenous groups, and involved frontier conflict, government policy and missionary programs that led to the expatriation of Aboriginal people from their tribal lands. Nonetheless, there remain some strongholds of traditional Aboriginal cultural practices in northern and central Australia. Of particular importance is Arnhem Land, which occupies the large northeastern coastal region of the Northern Territory and was never effectively invaded by Europeans^10^. In this region, many groups remained on their clan estates, sustaining their culture and fire management practices^11^. These enduring coupled human and natural systems are of paramount importance in understanding Indigenous fire regimes in Australian forest and savanna ecosystems, and more generally globally.

### Indigenous fire practices and pyrodiversity

A key concept in fire ecology is ‘pyrodiversity’, which emphasizes the linkage between fire regimes and the spatio-temporal patterns of fires and biodiversity^12,13^. It has long been maintained that traditional Aboriginal fire management by Australian Aboriginal people created fine-grained burn mosaics, and that this benefited biodiversity^6^, although there are few examples of this process. One prime example comes from the Western Desert region, where Aboriginal patch burning has been associated with increased abundance of prey items^14^. Another is traditional Aboriginal fire management in Arnhem Land, consisting of spatially-patchy, low-intensity fires, which are mostly set in the second half of the austral winter dry season^15,16^. In addition to clearing undergrowth to make travel on foot and camping easier, a key motive for burning is to provide macropod game (kangaroos and wallabies) with areas of nutritious resprouting grass^17,18^.

The demographic structure of stands of the Gondwanan conifer *C. intratropica* is well-recognised as a bio-indicator of pyrodiversity in savanna landscapes across the Australian monsoon tropics^16,19–26^. *C. intratropica* has termite-resistant timber, so dead stems can persist in the landscape for many decades, making the changes in the spatial distribution and population structure of the species very conspicuous^19^. Population declines have been attributed to the breakdown of traditional Aboriginal fire management during the twentieth century^19^. *C. intratropica* can survive low-intensity litter fires because adults and juveniles have thick, fire-resistant bark^27,28^, but more intense grass-fires typically partially defoliate adults and kill juveniles (seedlings and saplings), causing a severe recruitment bottleneck^27,29^.

## Objectives

The primary motivation for our work is to understand the ongoing transition from traditional Aboriginal fire management to contemporary fire regimes using *C. intratropica* as a bio-indicator of fire regime change^16,19,26,30^. This study is anchored on a contrast between two *Eucalyptus tetrodonta* savannas that occur on level, deep sandy soils: (a) the Dukaladjarranj estate in Arnhem Land, which has been continuously occupied, and (b) similar sand sheet savannas in Kakadu National Park which were depopulated in the early twentieth century. Previous studies have also contrasted the section of the Arnhem Plateau in Kakadu National Park with the Dukaladjarranj estate^15,18,21,30^.

Dukaladjarranj is the traditional estate of a *Kune*-speaking (a dialect of the *Bininj Kunwok* language) clan in central Arnhem Land^31^. This is one of the few Australian Aboriginal clan estates where colonialism did not significantly disrupt occupancy by the traditional custodians, other than a brief hiatus in occupancy in the mid-twentieth century^11,32^. This estate has been the subject of a multidisciplinary research program which commenced in 1997^11^. Since 2006, a broad-scale fire management program using aerial incendiaries has been implemented on the estate as part of a greenhouse gas emissions abatement program^33^. Previous studies have noted the good health of ecosystems at Dukaladjarranj, and *C. intratropica* populations in particular^11,21,26^.

Using dendroecology and population monitoring, we track demographic changes in *C. intratropica* on the Dukaladjarranj estate, spanning the period before and after the imposition of broad-scale fire management that commenced in 2006. We then contrast the Dukaladjarranj estate with adjoining, environmentally similar lands in Kakadu National Park on the western edge of the Arnhem Plateau, where traditional Aboriginal fire management has been more severely disrupted, due to depopulation in the mid-twentieth century. In 2007, Kakadu National Park commenced a fire management program designed to reduce the extent and frequency of late-dry season fires, which are widely understood to degrade biodiversity on the plateau. The geographic contrasts are based on extensive surveys of *C. intratropica* population structures, grass fuel loads and remote sensing analysis of fire regimes. We combine these findings with previously published studies from Dukaladjarranj and the ecology of *C. intratropica* more generally, to develop a conceptual model of how *C. intratropica* populations have changed following the breakdown of traditional Aboriginal fire management.

## Geographic and ecological context

### The Arnhem Plateau ‘Stone Country’

The Arnhem Plateau has an ancient human history with archaeological evidence dated at 65,000 years BP^34^. Molecular analyses suggest that individual language groups have had extremely strong fidelity to their clan estates (‘country’) since the Late Pleistocene^34,35^. During the early twentieth century, the great majority of Aboriginal people on the Arnhem Plateau, including within present-day Kakadu, migrated to surrounding lowland settlements and cattle ranches, buffalo hunting camps, small mines and missions. This trend partially reversed in the late twentieth century with the establishment of small settlements or ‘outstations’ on the northeastern edge of the plateau^32^. The development a major Uranium mine to the west of the Arnhem Plateau in the late 1970s led to the creation of Kakadu National Park, which is jointly managed by Australian Government and Aboriginal traditional custodians^36^. Since the early twenty-first century Kakadu has instituted fire management programs on the Arnhem Plateau^37^.

**Figure 1:**
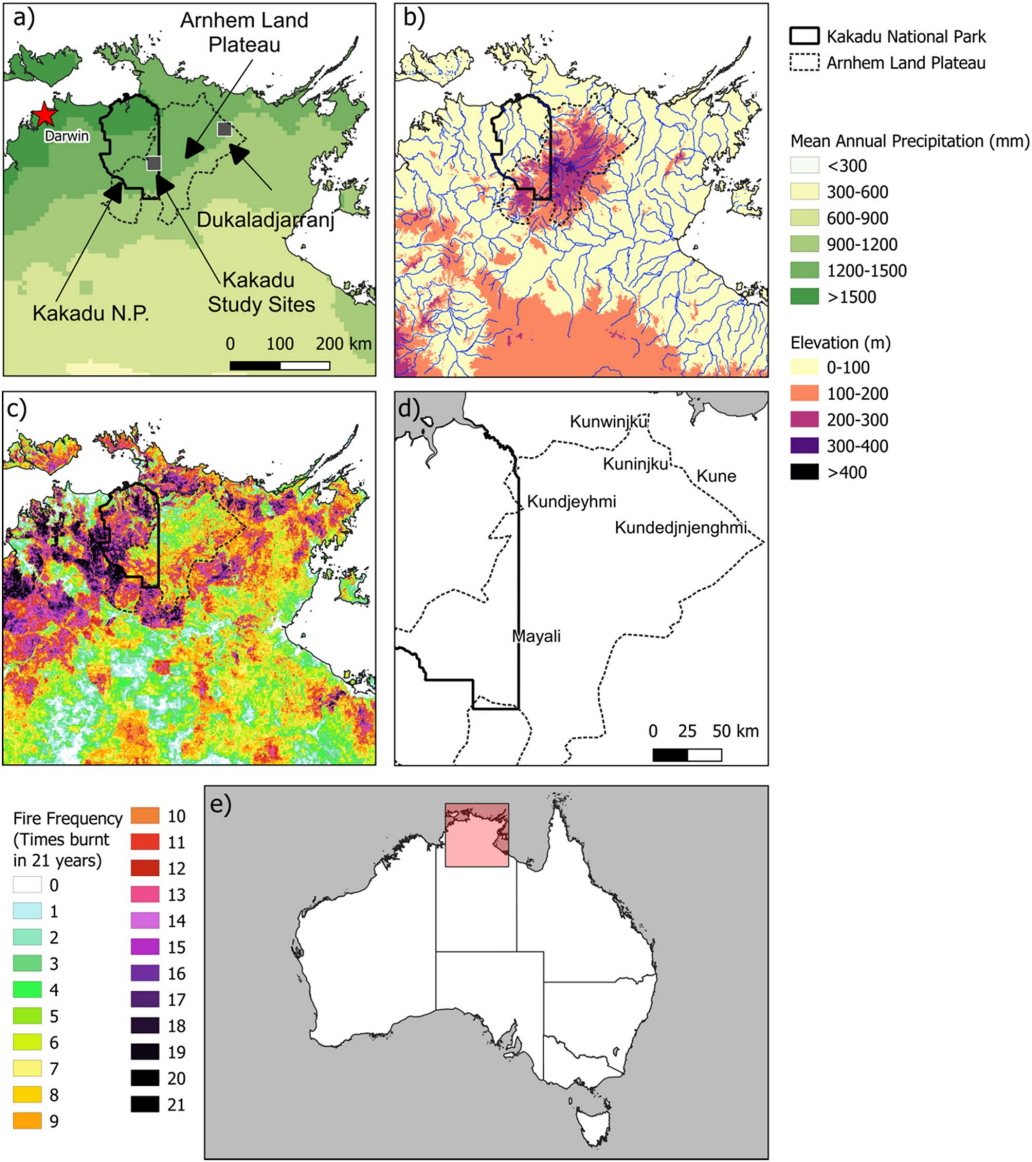
Arnhem Plateau study setting in the northern part of the Northern Territory, Australia. (**a**) Location of study sites in Kakadu National Park and the Dukaladjarranj Aboriginal clan estate in central Arnhem Land. Shading shows the broad rainfall gradient. (**b**) Elevation and major drainages. (**c**) Number of times burnt in 21 years (2000–2021, inclusive), according to the MODIS satellite record. (**d**) Major language groups across the Arnhem Plateau^31^. (**e**) Australian continental geographic context of the study region. The boundaries of Kakadu National Park and the boundary of the Arnhem Plateau (defined by outcropping sandstone^80^ are indicated in panels (**a**–**d**).

The Arnhem Plateau is characterized by extensive and deeply weathered Proterozoic sandstone, locally known as ‘stone country’ (Fig. 1). The plateau rises several hundred meters above flat, deeply weather laterite coastal plains with a steep escarpment to the north and northwest but the plateau surface gradually intergrades with the lowlands on its eastern and southern edges^38^. Some of Australia’s largest volume rivers debouch northward from the plateau, feeding highly productive estuarine freshwater floodplains before discharging into the Arafura Sea. The climate is strongly monsoonal, with over 1000 mm of mean annual rainfall concentrated in the austral summer months (December to February). The months of May to September are virtually rain-free^39^.

The Arnhem Plateau is an Australian biodiversity hotspot, with numerous endemic and/or threatened plants and animals^40^. Vegetation cover includes sparsely-treed heathlands and hummock grasslands dominated by the endemic Australian genus *Triodia* on skeletal soils, and open forests and woodlands dominated by *Eucalyptus* spp., considered a form of savanna, on deep sandy soils formed on sand sheets^15^. Embedded within the eucalypt savanna are scattered individuals and patches of *C. intratropica*. Marked rainfall seasonality favors very frequent fires. Humans are the most important ignition source, although lightning can be a common cause in the transition from the dry season to the wet season (October and November), when rain-free convection storms are common^41,42^.

It is well recognized that fire regimes in unpopulated areas of Arnhem Plateau have damaged the region’s biodiversity values^37,43,44^. Rectifying this has been the primary motivation for extensive fire management in Kakadu National Park, which systematically commenced in 2007^37^. Frequent (< 5 year) early dry season burning, set by aerial ignitions, in specifically targeted areas, is undertaken to impede late dry season fires, lit by either humans or lightning^37^. Concurrently, a broad-scale fire management program was implemented in western Arnhem Land in 2006 to generate tradeable carbon credits by reducing greenhouse gas emissions by increasing the area burnt in the early dry season, and thereby reducing the area burnt by late dry season fires^33^. The basis for this management approach is because early dry season fires generally have lower intensity and less complete combustion, which reduces greenhouse gas emissions compared with late dry season fires^33^. Although the primary motivations of these institutionalized fire management interventions vary between Kakadu National Park (primarily biodiversity conservation) and western Arnhem Land (involving carbon credits, Aboriginal employment, capacity building in land management, and biodiversity conservation) they share many similarities, as both are based on reducing the extent of late dry season fires, which are perceived as substantially more detrimental to biodiversity than early dry season fires^33,44^. However, Corey et al.^44^ have cautioned that substitution of late season fires with burning in the early dry season does not necessarily increase or maintain biodiversity, especially for organisms that require areas of unburnt habitat.

### Callitris intratropica

*Callitris intratropica* belongs to the Callitroideae, a southern hemisphere subfamily of the cypress conifer lineage (family Cupressaceae), which has an evolutionary history of over 200 Myr, predating the separation of Gondwana and Laurasia^45^. Unlike most other southern hemisphere conifers that went extinct during the mid-Cainozoic aridification of Australia, the *Callitris* clade was able to adapt to both drought and periodic landscape fire^45^. *C. intratropica* trees have thick insulating bark that protect the cambium from surface fires but has very weak post fire resprouting^27^. While many close relatives are fire-adapted, with fire-cued seed release from serotinous, robust woody cones, *C. intratropica* is classified as a fire-intolerant obligate seeder because it lacks a long-lived soil or aerial seedbank^45,46^. *C. intratropica* is an extreme xerophyte, like most other members of the genus, giving it a competitive advantage in fire-sheltered sites in drought-prone and seasonally dry environments^47^. Genetic analyses suggest *C. intratropica* populations were little affected by Pleistocene environmental change, including the Aboriginal landscape burning which commenced in the Late Pleistocene^48^. This is in contrast to closely related southern and central Australian species that lost genetic diversity as their ranges contracted due to intense Late Pleistocene aridity^48^.

The intense rainfall seasonality of the Australian monsoon tropics results in *C. intratropica* trees having pronounced growth rings, the widths of which scale to the duration of the wet season^49^. Previous dendrochronological research indicates this species is long-lived, with many specimens living for at least 200 years^50,51^. Large stems > 80 cm in diameter^26^ are likely older than 400 years, based on average annual growth rates^52^.

The species is wind pollinated, produces irregular seed crops and, once mature, cones release seeds that are wind dispersed, with dispersal typically restricted to about 60 m from the tree, with pronounced seed shadows and asymmetrical clumps of dense regeneration^53,54^. Whether fertility and fecundity declines with age is unknown, although intense fires that damage canopies are known to reduce seed output^30,55^. Additionally, isolated individuals could be at risk of inbreeding depression that affects seed viability^56^.

## Results and discussion

### Fire regimes

**Figure 2:**
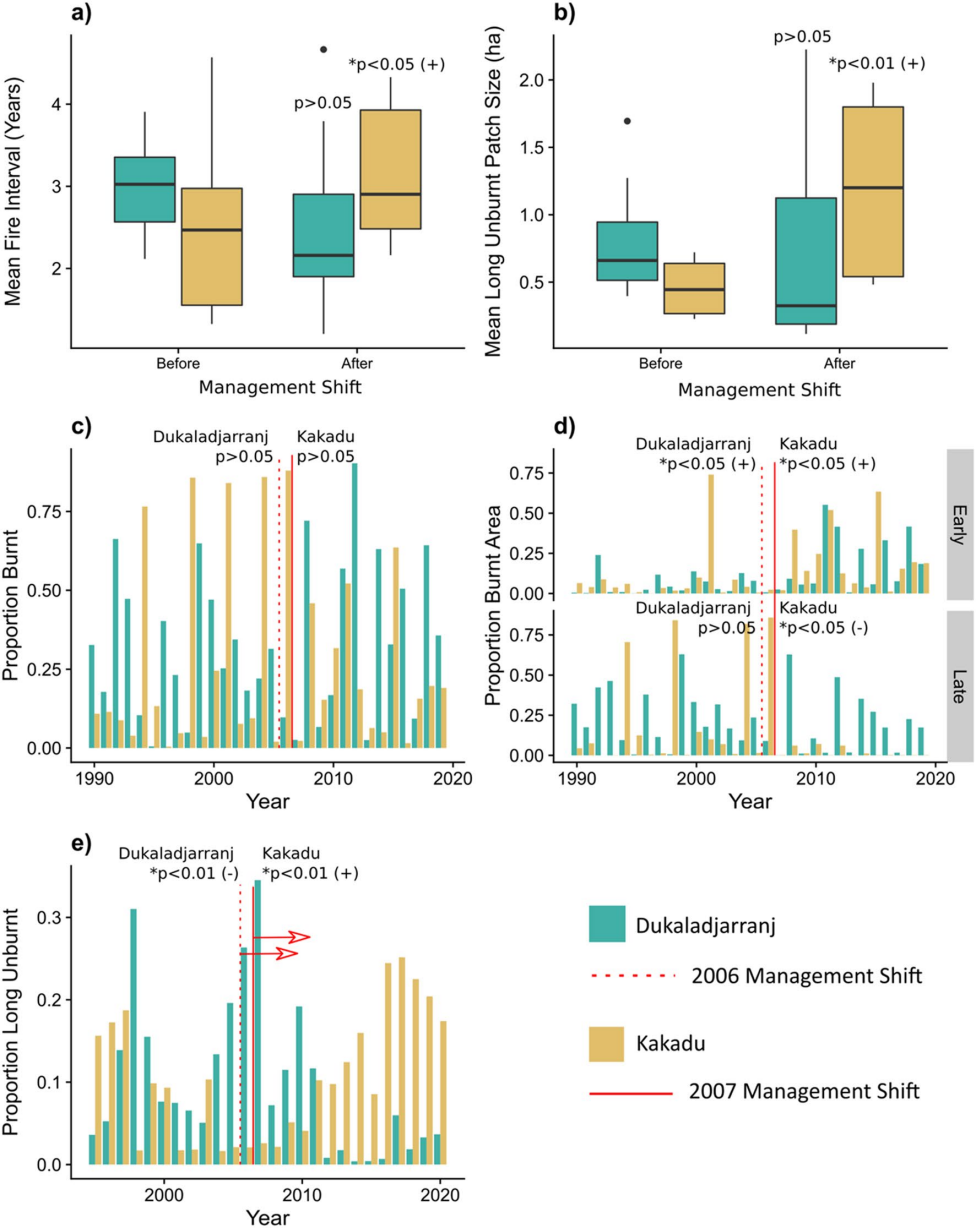
Trends in fire regimes on *Eucalyptus* sand sheet savannas in Kakadu National Park and the Dukaladjarranj Aboriginal estate in central Arnhem Land between 1990 and 2020. The analysis is based on analysis of differential normalized burn ratio calculated from seasonal LANDSAT surface reflectance composites for sand sheet savannas in areas that were sampled by transects in Arnhem Land and Kakadu (see Supplementary Materials Fig. S1 and Fig. S4). Note that because the areas sampled in Arnhem Land and Kakadu National Park are very different (407 vs. 3592 ha) all mapped areas are expressed as proportions of the sample domains. The start for the fire management interventions in central Arnhem Land (2006) and Kakadu National Park (2007) are indicated with dashed and solid red lines respectively. In the case of panel e there is a 5-year lag indicated by horizontal arrows. (**a**) Box and whisker plots of the mean time since pre- and post- fire management interventions in Kakadu National Park and Dukaladjarranj. Statistical differences are shown for linear model of mean time since fire against period. (**b**) Comparison of mean long unburnt (> 5 years) patch size for Kakadu National Park and Dukaladjarranj pre and post fire management interventions in Kakadu National Park and Dukaladjarranj. Statistical differences are shown for linear model of log mean patch size against period. (**c**) Trends in the proportion burnt in Kakadu National Park and Dukaladjarranj. Statistical differences are shown for linear model of arc-sine square root transformed annual proportion burnt against period. (**d**) Trends in the proportion area burnt in early (March to June inclusive) and late (July to October inclusive) dry season for Kakadu National Park and Dukaladjarranj. Statistical differences are shown for linear model of arc-sine square root transformed season proportion burnt against period. (**e**) Trends in the proportion area long unburnt (> 5 years) for Kakadu National Park and Dukaladjarranj. Statistical differences are shown for linear model of arc-sine square root transformed proportion long unburnt against period.

Our remote sensing comparison of fire regimes (see "Materials and Methods" Section) on sand sheet savannas revealed significant differences between Kakadu National Park and Dukaladjarranj in Arnhem Land, both before and after the fire management interventions that commenced in 2007 and 2006 respectively (Fig. 2). In Kakadu, the fire management intervention has had the following statistically significant effects: (1) increased the time interval between fires; (2) increased the area and mean patch size of longer unburnt vegetation (> 5 years); and (3) switched the predominant season of fire from the late (August–November) to early dry season (May–July) (Fig. 2). Critically, however, it has not significantly changed the total proportion of landscape burnt each year (Fig. 2). These trends are broadly consistent with previous analyses of the effects of early dry season fire management^33,37,43^. At Dukaladjarranj there was no statistically significant change in the total proportion of the landscape burnt each year, the interval between fires, or the patch size of longer unburnt vegetation (> 5 years) (Fig. 2). The area burnt by early season fires, however, has increased, and the proportion of long unburnt vegetation has decreased (Fig. 2). These changes can be explained by the adding together institutionalized fire management in the early dry season with traditional Aboriginal fire management that is concentrated in the second half of the dry season^15,57,58^. Ethnographic^18^ and ecological research^17^ has shown that a key motivation for Aboriginal people in central Arnhem Land to burn in the late dry season is to provide nutritious resprouting vegetation for macropod game species. Institutional managers, by contrast, prefer early dry season burning as a pragmatic means of controlling more intense and extensive late dry season fires, by creating fuel breaks and removing heavy grass fuels^15,57^.

### *Callitris intratropica* demographics

**Figure 3:**
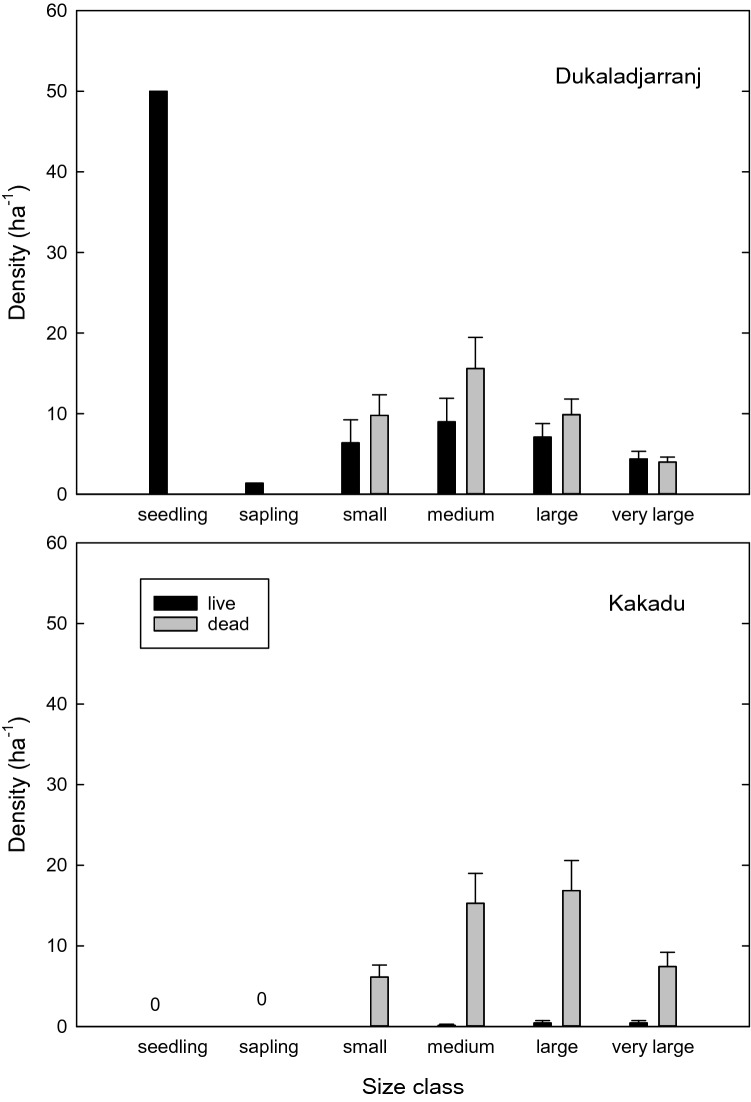
Mean (and standard error) densities of live and dead *Callitris intratropica* seedlings, saplings, and trees in *Eucalyptus* sand sheet savannas in transects in Kakadu National Park and the Dukaladjarranj Aboriginal estate in central Arnhem Land. Transects sampled a total of 8 ha in Kakadu National Park and 10 ha at Dukaladjarranj Aboriginal estate (Figures S1 and S2). None of the Kakadu transects contained any saplings or seedlings. Size classes are defined as: seedlings (< 0.1 m height), saplings (0.1–2.2 m height), small trees (≥ 2.2 m tall and < 10 cm diameter at 1 m height (D_1.0_)), medium trees (10–20 cm D_1.0_), large trees (20–30 cm D_1.0_) and very large trees (≥ 30 cm D_1.0_). Dead seedlings and saplings were not recorded as they break down very quickly. Assuming an average growth rate of 2.5 mm/year^30^, and that it takes seedlings 18 years to reach 1 m height (Eq. 3) the size classes can be converted to indicative age classes as follows: small trees are < 60 years old; medium trees are 60 to 100 years old; large trees are 100 to 140 year old, and very large trees are > 140 years old. As the measurements were made in 2016, the size classes capture trees that established since the mid nineteenth century. It is important to note there is uncertainty when the dead trees established or died.

At Dukaladjarranj, surveys (see "Materials and Methods" Section) on two intersecting transects sampling a total of 10 ha demonstrated abundant living *C. intratropica* (mean and standard error: 26.9 ± 7.2 ha^−1^) and sparse dead stems (Fig. 3) (see "Materials and Methods" Section). This was in stark contrast to Kakadu National Park, where we surveyed *C. intratropica* populations on sand sheet savannas using 50 m wide transects sampling four areas in Kakadu (total area 8 ha and length 3.2 km), and found no *C. intratropica* seedlings or saplings, with very low density of living trees (mean and standard error: 0.9 ± 0.5 ha^-1^) yet an abundance of dead trees (Fig. 3). The counts of dead and live trees combined in the larger size classes were similar between the Kakadu and Arnhem Land sites, indicating that these sites originally had similar living populations of *C. intratropica* (Fig. 3). These data reinforce previous surveys on the Arnhem Plateau and elsewhere in northern Australia that report severe recruitment bottlenecks in *C. intratropica* populations^19–23,26^.

**Figure 4:**
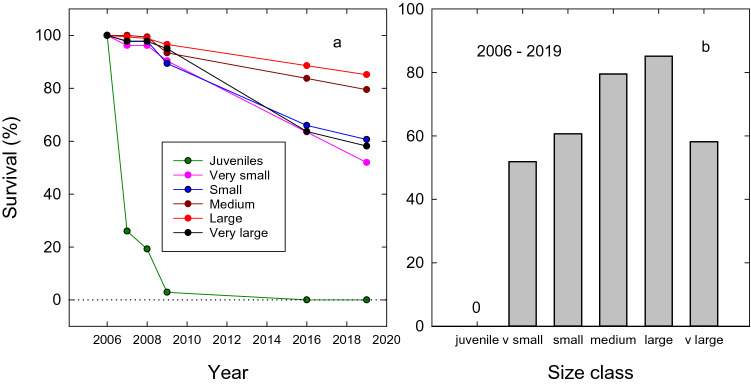
Percentage survival of the various size classes of *Callitris intratropica* at the Dukaladjarranj Aboriginal estate in central Arnhem Land: juveniles < 1.5 m tall (n = 385); very small trees > = 1.5 m tall and < 5 cm diameter at 1 m (D_1.0_, n = 52); small trees 5–10 cm D_1.0_ (n = 94); medium trees are 10–20 cm D_1.0_ (n = 166); large trees 20–30 cm D_1.0_ (n = 175); and very large trees > = 30 cm D_1.0_ (n = 220). (**a**) Survival through time, and (**b**) survival to 2019 by size class, 13 years after the trees were tagged.

**Figure 5:**
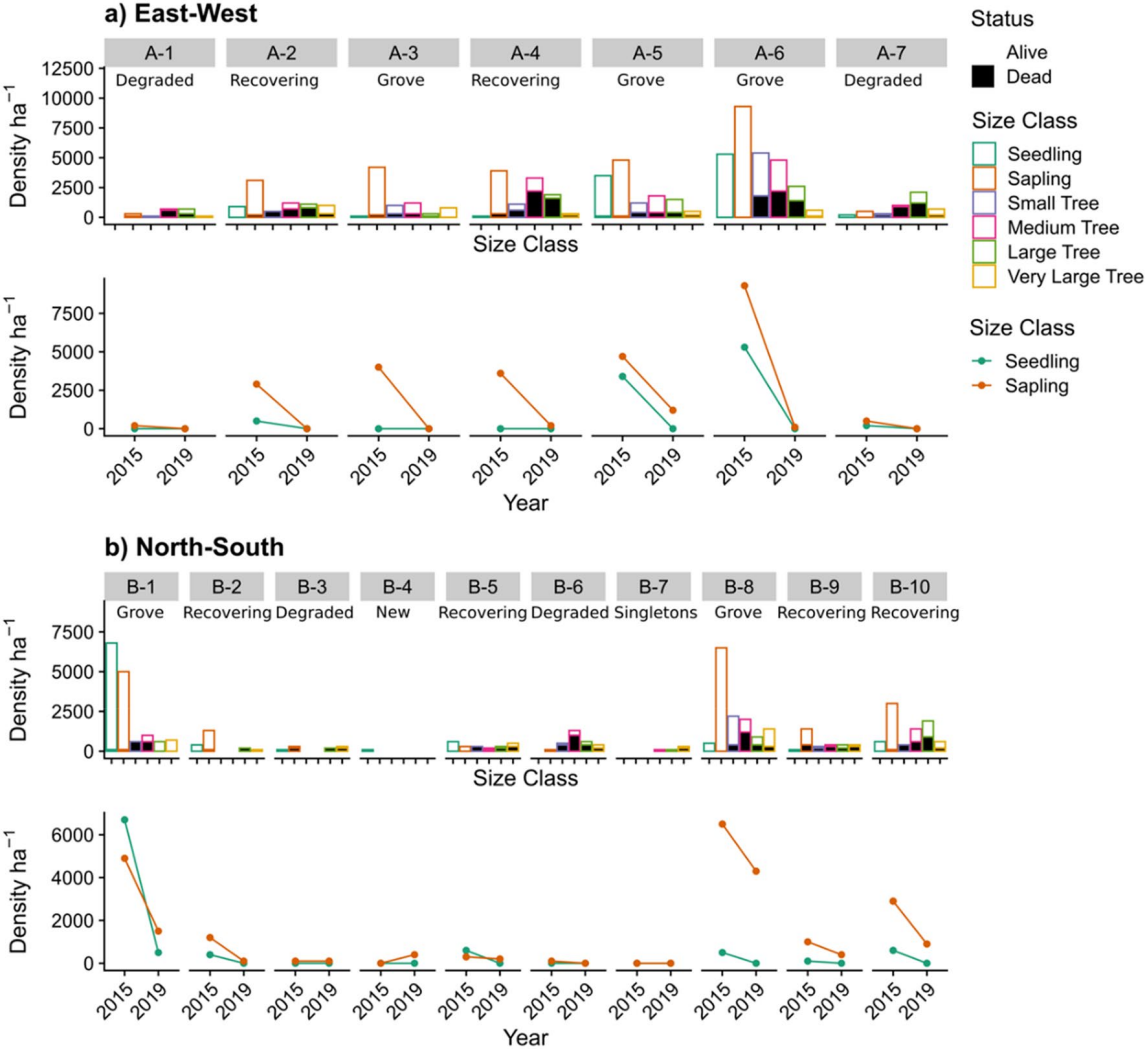
Size class distributions and status of *Callitris intratropica* stands in 100 m segments of the two transects (**a**,**b**) at the Dukaladjarranj Aboriginal estate in central Arnhem Land. Also shown are changes in the density of seedlings and saplings between 2015 and 2019 (Fig. S4). Seedlings (green) and saplings (orange) are respectively defined as < 0.1 m tall and 0.1 to 2.2 m tall. Seedlings, saplings and trees were counted within bands 2 m, 25 m and 25 m either side of the transect midline, respectively. Size classes are defined as: seedlings (< 0.1 m tall), saplings (0.1 to 2.2 m tall), small trees (≥ 2.2 m tall and < 10 cm D_1.0_), medium trees (10 to 20 cm D_1.0_), large trees (20 to 30 cm D_1.0_) and very large trees (≥ 30 cm D_1.0_). Stand status in each segment was defined as healthy groves (multiple live trees in most size classes, well outnumbering dead trees); degraded (a few live trees in larger size classes, with a high proportion of dead trees and few saplings or seedlings); recovering (similar to degraded, except with multiple seedlings and saplings); singletons (very few trees, mostly alive); and new (seedlings and/or saplings only).

The development of the recruitment bottleneck at Dukaladjarranj, following the imposition of broadscale fire management, is clearly apparent in a 13-year (2006–2019) longitudinal study of the survival of 1172 *C. intratropica* individuals at Dukaladjarranj (Fig. 4) (see "Materials and Methods" Section). No juveniles (< 1.5 m tall in 2006) survived to 2019, and only 52% of the very small trees (≥ 1.5 m tall and < 5 cm D_1.0_) survived (Fig. 5a). By comparison, 70% of all trees ≥ 1.5 m tall survived over the 13-year sampling period. Thus, the 0% annualized survival of juveniles compares with 97.3% annualized survival rate of adult trees (> 1.5 m tall) overall. The highest annualized survival was 98.8% for large trees (20–30 cm D_1.0_), with trees larger than this appearing slightly more vulnerable to fire, with 95.9% annualized survival. We found additional evidence of a recruitment bottleneck developing at Dukaladjarranj in the surveys on two transect between 2015 and 2019, which showed very high juvenile mortality (Fig. 5a,b), including substantial losses from multi-aged groves of *C. intratropica* that have previously been shown to exclude fire under mild to moderate fire weather conditions^59^. This is in in contrast to previous studies at Dukaladjarranj that showed frequent recruitment of juveniles into the larger size classes^11,21,26^. Collectively, our results reinforce the conclusion of previous demographic studies that the senescence and eventual collapse of *C. intratropica* populations growing on sand sheet savannas in Kakadu National Park is the culmination of a gradual demographic process involving the high mortality of juveniles and the multi-decadal attrition of adult tree density and associated loss of fecundity^25,30,56,60^. The observed bottleneck in juvenile recruitment at Dukaladjarranj and population declines of adults suggests this terminal demographic trajectory is developing there.

### Grass fuel loads

**Figure 6:**
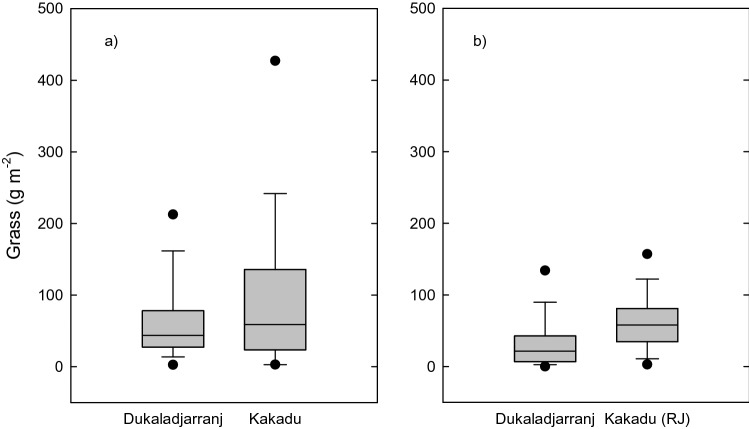
Grass biomass sampled in transects on *Eucalyptus* sand sheet savannas in Kakadu National Park and the Dukaladjarranj Aboriginal estate in central Arnhem Land. (**a**) Grass biomass was sampled in eight 0.25 m^2^ quadrats per 100 m segment of transect. Total transect length was 2.0 km at Dukaladjarranj and 3.2 km in Kakadu (see "Materials and Methods" Section). (**b**) Box plots showing loads of grass and litter fuels sampled in four 0.25 m^2^ quadrats in circular plots (10 m radius) surrounding living *C. intratropica* trees at Round Jungle (RJ) in Kakadu National Park and Dukaladjarranj (Fig. S1). Boxes span the interquartile range, the solid line indicates the median, the whiskers the 10th and 90th percentiles and solid circles, 5% and 95% outliers.

In addition to this marked difference in stand structure of *C. intratropica*, Kakadu and Dukaladjarranj differed markedly in their grass layer. Our field traverses (see "Materials and Methods" Section) showed grass biomass averaging 112 g m^−2^ in our Kakadu transects, compared with 71 g m^−2^ in the Dukaladjarranj ones (Fig. 6a). These differences were driven by higher maximum fuel loads in Kakadu: the 0.90 quantile regression strongly supported regional differences, with modelled loads higher in Kakadu by 77 g m^−2^ (95% confidence interval [CI] 35–167 g m^−2^). The modelling provided strong support for a difference between Kakadu and Dukaladjarranj, with AIC for the model including ‘region’ 21.6 units lower (indicating a much better model) than the intercept-only model. The 0.50 quantile regression showed a smaller difference between regions, predicting fuel loads higher in Kakadu by 15.2 g m^−2^ (95% CI 19–39 g m^−2^) and weaker support, with the AIC of the regional model being 3.2 lower than that of the intercept-only model.

More detailed surveys (see "Materials and Methods" Section) of fuels surrounding living *C. intratropica* trees found grass fuel loads were almost twice as high in a remnant population near Round Jungle in Kakadu^52^ than at Dukaladjarranj (average 64.4 vs 35.6 g m^−2^ respectively) (Fig. 6b). This difference had clear statistical support (AIC of the regional model was 10.5 lower than the intercept-only model for the 0.90 quantile, and 56.5 lower for the 0.50 quantile).

Previous work has also shown that healthy stands of *C. intratropica* (with few dead trees and abundant juveniles) have low grass biomass, and hence are less likely to support intense fires than degraded stands (with abundant dead trees and no juveniles), which tend to have high grass biomass^54,59^. Experiments and field observations have linked grass biomass with recruitment bottlenecks and adult tree mortality in C. *intratropica*. Juveniles of this species have some resistance to litter fires, but they are damaged and often killed by grass fires, which burn at higher intensities and have taller flames^19,29,60^. Fire experiments have also demonstrated that when grass fuel loads are high, fires can readily partially or completely defoliate *C. intratropica* canopies, reducing tree health or killing stems^27^.

### Fire mosaics and regeneration of *Callitris intratropica*

The apparent paradox of a tree sensitive to grass fires persisting in a tropical savanna environment can be explained by the regeneration patch dynamics of the species. Population modelling suggests that *C. intratropica* groves are transitory landscape features associated with changes in the frequency and severity of landscape fires^30^. A regime of small, patchy fires promotes *C. intratropica* persistence in tropical savanna both by reducing the frequency of high intensity fires and increasing the abundance of long-unburnt habitat patches^61^. Closed-canopy groves of *C. intratropica* are able to exclude, or substantially reduce the intensity of, fires by suppressing fine fuels, particularly grass biomass, allowing prolific regeneration within the fire-protected stands and providing habitat for other fire-sensitive plant species^59^.

**Figure 7:**
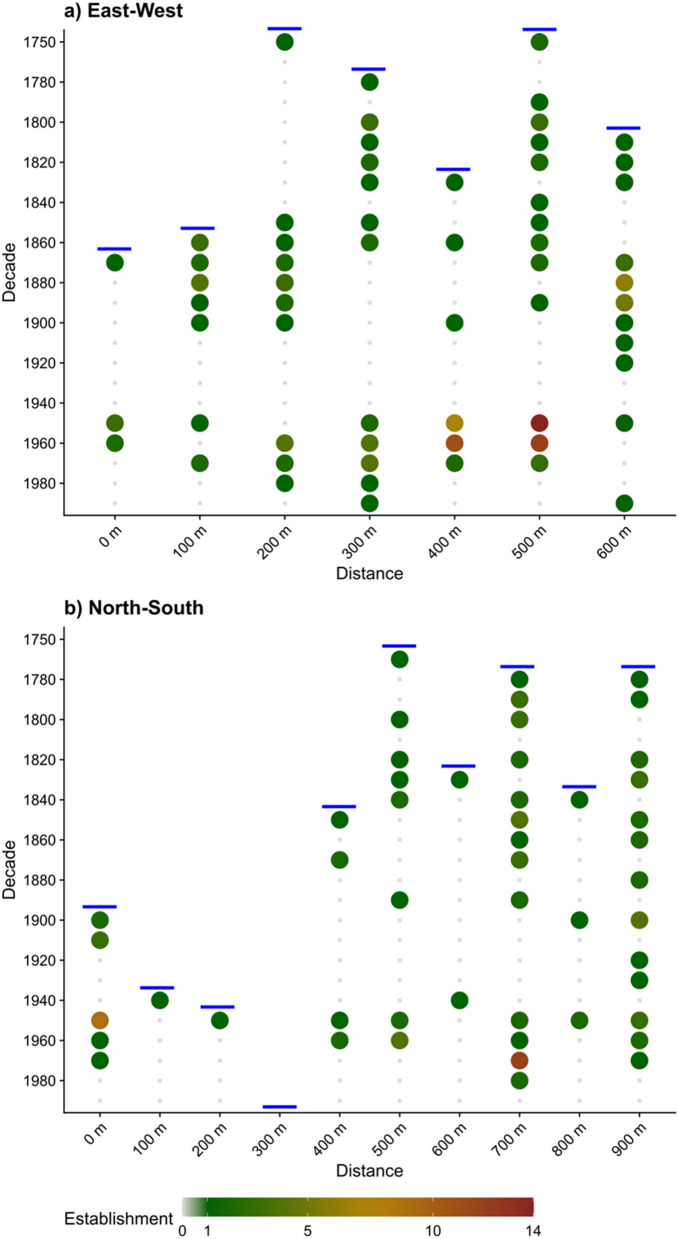
Establishment dates determined by dendrochronological analysis of sampled *Callitris intratropica* trees on two transects (**a**,**b**, see Fig. 6ab and S4) at Dukaladjarranj Aboriginal estate in central Arnhem Land (Fig. S4). Shading denotes the number of trees that established in each decade. The blue horizontal lines denote the limit of the dendrochronological record for each section on the transect.

These spatio-temporal inferences are supported by our demographic and dendrochronological analysis (see "Materials and Methods" Section) of *C. intratropica* establishment on two transects at Dukaladjarranj (Fig. 7a,b). The sub-hectare spatial patterning of *C. intratropica* regeneration is clearly apparent, with groves containing seedlings and saplings separated by areas with low densities of older trees without any regeneration. Dendroecological analyses shows that regeneration has been continuous, albeit spatially variable, since at least 1750, noting there are fewer samples at the limit of the dendrochronological record. It is plausible that the absence of recruitment in the middle of the twentieth century reflects a hiatus in the occupancy of the estate by the Aboriginal traditional custodians^32^. Overall, these patterns corroborate prior research that demonstrated that *C. intratropica* persists in frequently burnt savannas in closed canopy groves, which tend to exclude fire, and which are spatially and temporally dynamic; lone trees are more exposed and vulnerable to fire, have lower survival, and do not provide suitable microhabitat for the establishment and survival of juveniles^30^.

**Figure 8:**
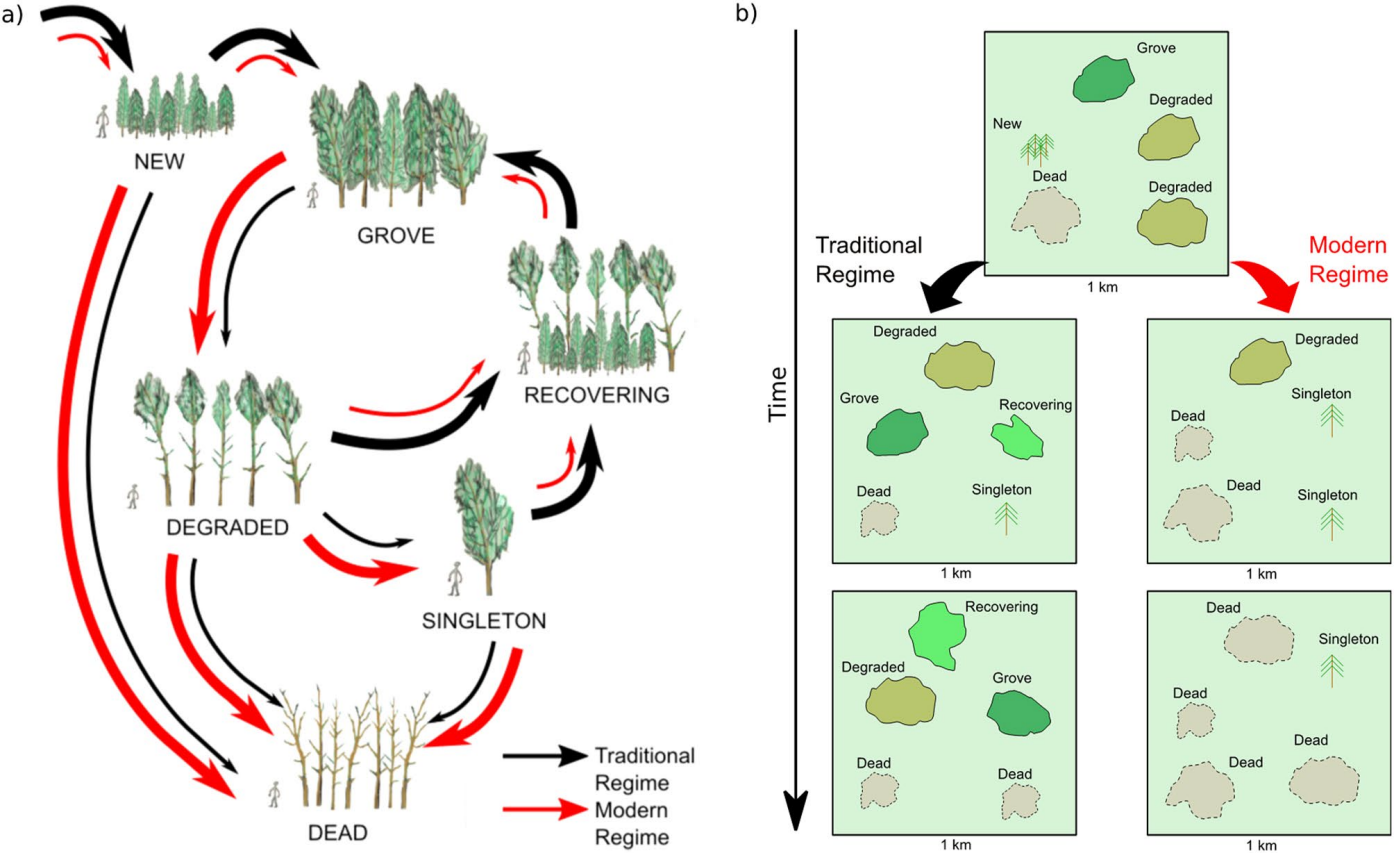
Conceptual model of the stand and spatial dynamics of *Callitris intratropica* on sand sheets on the Arnhem Plateau, based on a synthesis of results from this and previous publications (see main text for details)**.** (**a**) Small *C. intratropica* groves (stands) fluctuate in size and structure in response to fire damage which affects the ability of groves to exclude flammable grasses. The incursion of grasses into the stands allows fires to repeatedly burn into the groves. Once degraded, groves often die or are reduced to single isolated trees within a relatively short period of time. Singleton trees eventually die or, if fire frequency is reduced, can lead to the development of a new fire-excluding grove. Seed dispersal can also lead to new groves if seedling cohorts are able to establish in areas free from fire for several decades. In the diagram, the width of each arrow reflects our approximation of likely probability of the transition between the various states. (**b**) Under traditional Aboriginal fire regimes, the stand dynamics result in an ever-shifting mosaic of groves and singleton trees on fire exposed *Eucalyptus* sand sheet savannas. Under the modern fire regime of Kakadu National Park, the frequency and intensity of fires has increased such that the groves have been destroyed and most singleton trees on sand sheets have died. This is a gradual process that commenced in the twentieth century following the breakdown of traditional Aboriginal fire management. We suspect that complete elimination of *C. intratropica* on fire-exposed sand sheets is likely in the next few decades under current fire regime.

We present a conceptual model that draws together the previous research findings on *C. intratropica* combined with those of this study (Fig. 8). The model suggests that the patch dynamics of *C. intratropica* that was sustained by traditional Aboriginal fire management is being overwritten my modern fire regimes, thereby explaining the development of a population bottlenecks at Dukaladjarranj and the landscape scale collapse of *C. intratropica* populations in Kakadu National Park.

### Management implications

A program of frequent early dry season burning initiated in 2007 on the sandstone plateau in Kakadu National Park to create firebreaks has been shown to increase the area of less frequently burnt vegetation^37^; however, this change in fire regime has not led to a recovery of *C. intratropica* populations we sampled. Likewise, ongoing early dry season burning programs in western Arnhem Land initiated in 2006 have been acknowledged as unable to reverse declines of plants and animals that require infrequently burnt habitat^43^. At Dukaladjarranj estate, we found this management regime is associated with an increased frequency of fire in the early dry season and a decline in the proportion of long-unburnt vegetation, apparently leading to a recruitment bottleneck of *C. intratropica*.

Collectively, this and other studies highlight the difficulty in reversing the cascading ecological effects following the cessation of traditional Aboriginal fire management, which maintained a very fined-grained mosaic of low-intensity fires, low grass fuel loads, and habitat for fire-sensitive plants and animals, including providing nutritious forage for game species. The loss of traditional Aboriginal fire management has resulted in an ecological state-shift associated with a grass‒fire cycle^62,63^, causing frequent, large and high-intensity fires^27^. Improving biodiversity outcomes from fire management demands greater attention to creating pyrodiverse patterns that more closely resemble those achieved by Indigenous people managing fire for multiple purposes. This probably requires finer-scale, more spatially dispersed, ground-based burning rather than use of aerial incendiaries which dominate institutional programs, and a reduction in fire frequency, thereby reducing grass abundance. More broadly, this study highlights the global challenges of implementing fire regimes that can conserve biodiversity in the Anthropocene^64^. Surviving, and historically-documented, Indigenous management practices provide important insights and inspiration for this critical task^61,65–68^, However, we show that these practices are neither simple to restore, easy to replicate by colonial institutions, nor likely to readily reverse the biodiversity declines initiated by colonial disruption of refined socio-ecological traditions of fire management.

## Conclusions

Colonial processes initiated in the past have left long-term legacies in the present and for the future. Globally, many of these processes happened far enough in the past to make it difficult to disentangle colonial actions from the loss of Indigenous management practices^7^. Here we show the ongoing effects of colonialism in Arnhem Land where traditional fire management regimes were negatively affected by depopulation of most traditional Aboriginal estates in the early twentieth century. Nonetheless, a few estates such as the one we have studied maintained traditional fire management practices. In the twenty-first century institutionalized fire management programs have been established to reduce fuels, sustain biodiversity and reduce carbon emissions, goals largely defined by the colonial powers^69^. Because population relocation and the shift towards institutionalized land management happened so recently in our broad study region (in the past 100 years), we have been able to identify the chronology of ecological consequences of first removal of Indigenous traditional burning and then replacement with an altogether different form of fire management. We have shown these programs can overwrite the remaining traditional fire regimes where they persist, but are unable to restore them where they have been lost. Restoring the lost ecological functions of people^70^ would be effective, but only in those places that have not yet crossed a threshold beyond which biodiversity, including *C. intratropica* populations, cannot recover. The difficulty in reversing degradation of *C. intratropica* populations following changes in fire regimes revealed by this study suggests that some colonial legacies, in Arnhem Land and elsewhere, cannot be easily recovered by simply restoring Indigenous fire management practices. Managing such ecological hysteresis likely requires more comprehensive ecological restoration or rewilding interventions for Indigenous fire management to have desired outcomes^71^. Regardless, our work highlights how some cultural practices are necessary to maintain ecosystem resilience. We therefore support calls for greater investment in initiatives that give Indigenous communities greater self-determination and control over ecological management of ancestral lands to repair some of the social and ecological harms caused by centuries of colonialism^72^.

## Materials and methods

Fieldwork occurred at two locations on the Arnhem Plateau: at Dukaladjarranj, the estate of a *Kune*-speaking clan on the northeastern edge of the plateau in central Arnhem Land, and on the western edge of the plateau in Kakadu National Park (Fig. S1).

### Fire history analyses

The fire history of the Arnhem Plateau was reconstructed using NASA Landsat seasonal surface reflectance composite images^73^ from 1989 to 2020. Analysis was restricted to polygons drawn by manual satellite image interpretation bounding sand sheet vegetation units surrounding the transect locations in Kakadu National Park and Dukaladjarranj estate in central Arnhem Land, avoiding rocky outcrops and riparian vegetation (Fig. S2). In Kakadu National Park, 3592 ha of sand sheet savanna was sampled and 407 ha was sampled at Dukaladjarranj. Early dry season fire detections were based on the difference between March–May and June–August composites, while late dry season fires were based on the difference between June–August and September–November composites. For each seasonal pair of composites, an overall mean (1989–2020) differential normalized burn ratio (dNBR) was calculated^74^ and a cut-off value was identified and used to delineate burnt areas. It is important to note that in this savanna environment it is not possible to accurately map levels of fire severity^75^. Individual dNBR rasters were then generated for individual years within each seasonal pair, from which the all-year mean seasonal dNBR was subtracted, to increase contrast and remove static landscape features. Cells with dNBR greater than 0.75 were categorized as having fire present in the interval, with this threshold value chosen as it provided good visual agreement with lower-resolution burnt area polygons derived from the MODIS and VIIRS satellite-borne sensors, obtained from the North Australia and Rangelands Fire Information Service (https://firenorth.org.au/nafi). While Landsat composites lack temporal resolution and have limitations in obtaining cloud-free imagery early or late in the season^76^, they provide much greater spatial resolution to detect small unburnt patches (Fig. S3). Time since fire rasters were calculated for the period with at least five years of fire history available (1995–2020). The ‘landscapemetrics’ package in R^77^ was used to calculate the area of patches with time since fire greater than five years in each interval, and summary statistics of mean time since fire per pixel, and the early, late and total area burnt each yere were tabulated.

Linear regressions were performed to detect significant differences in fire regime pre- and post-fire management interventions for the two study areas, for the following variables: mean time since fire, mean long unburnt (> 5 years) patch size, annual proportion burnt (total, early and late) and annual proportion in the long unburnt class. Data were classified into pre- and post-intervention using 2006 as the threshold year for Dukaladjarranj, and 2007 for Kakadu, except for the test for proportion long unburnt, where threshold years of 2011 and 2012 were used instead to accommodate the lag before the long unburnt class was expected to change. Mean long unburnt patch size was log-transformed for analysis, and proportions were arc-sine square root transformed.

### Longitudinal mortality of *Callitris intratropica* at Dukaladjarranj

In 2006, 1171 *C. intratropica* individuals were tagged and geolocated at Dukaladjarranj: 385 juveniles (< 1.5 m tall); 52 very small trees (≥ 1.5 m tall and < 5 cm diameter at 1 m [D_1.0_]); 94 small trees (5–10 cm D_1.0_); 166 medium trees (10–20 cm D_1.0_); 175 large trees (20–30 cm D_1.0_); 220 very large trees (≥ 30 cm D_1.0_). In 2007, 2008, 2009, 2016 and 2019 the individuals were relocated, and their survival assessed. In instances where trees had fallen or been consumed by fire, a metal detector was used to locate tags where possible. Annualized survival (%) of individuals (≥ 1.5 m tall) was calculated as

1 $${\left(\frac{{N}_{2}}{{N}_{1}}\right)}^\frac{1}{y}\times 100$$

where N_1_ and N_2_ are the number of live individuals at the start and end of the monitoring interval, respectively, and y is the number of years in the monitoring interval^52^. All missing individuals were assumed to be dead.

### *Callitris intratropica* stand structures at Dukaladjarranj

In 2015, at Dukaladjarranj we established two transects, both 1000 m long and 50 m wide (25 m each side of a measuring tape representing the midline). The two transects were arranged perpendicular to each other in a cross-shape (Fig. S4), sampling a total of 10 ha. These transects were used for the dendrochronological study (see below) and the Dukaladjarranj stand structure analysis and fuel sampling.

In 2016, we measured diameter at one meter (D_1.0_) of living and dead *C. intratropica* trees > 2.2 m tall, as well as the height of live *C. intratropica* juveniles (0.1–2.2 m tall) and, within 2 m either side of the midline, small seedlings (< 0.1 m tall). The orthogonal distance from the midline of the transect to all *C. intratropica* trees, seedlings and juveniles was recorded using a sighting compass and ultrasonic hypsometer (Haglöf Vertex 5; Långsele, Sweden). The size class data were binned in twenty 100 m segments (0.5 ha each), with the total area sampled being 10 ha (Fig. S2). In 2019, we re-surveyed 1700 m of the transects (Fig. S1).

### *Callitris intratropica* stand structures in Kakadu National Park

In July 2016 we undertook field traverses through trackless country on the western edge of Arnhem Plateau in Kakadu National Park, searching for *C. intratropica* trees in *Eucalyptus tetrodonta* sand sheet savanna. We walked a total of 30 km, sampling three separate locations (Fig. S1). At each location we established transects comprised of segments 100-m long and 25-m wide (12.5 m either side of the midline measuring tape). These originated near the first *C. intratropica* tree (alive or dead) encountered on the field traverse, with subsequent 100-m segments placed until there were no more *C. intratropica* trees within 100 m of the last measured tree. The direction of each 100-m segment was adjusted to follow the line of trees and therefore maximize the transect length and number of *C. intratropica* trees captured in each stand. There were a total of 32 segments (each 0.25 ha), thus a total area of 8 ha was sampled. In each 100-m segment we measured diameter at one meter (D_1.0_) of all living and dead *C. intratropica* trees > 2.2 m tall and the height and basal diameter of *C. intratropica* juveniles between 0.1 and 2.2 m tall. We searched for live *C. intratropica* seedlings (< 0.1 m tall) within 2 m of either side of the midline of the transect.

### Grass fuel loads at Dukaladjarranj and in Kakadu National Park

In 2016, we collected grass and litter fuel samples from 8 regularly spaced 0.5 m × 0.5 m quadrats on the midline of each of the 20 100-m segments of the transects at Dukaladjarranj Aboriginal estate and the 32 100-m segments in Kakadu National Park, described above (Fig. S4). Samples were weighed in the field with a balance. Subsamples were then taken, weighed in the field and transported back to the laboratory where they were dried and reweighed for determination of moisture content. Fuel weights were averaged for the quadrats on each transect, and fuel dry weight estimated to determine differences between fuel load between Dukaladjarranj and Kakadu, noting there was very high variability in these data, especially in Kakadu. We were interested in both median and maximum grass loads, so we analysed grass biomass using quantile regression (0.90 and 0.50 quantiles), which is suited to heteroscedastic data, using the R package ‘quantreg’^78^.

We further investigated differences in the abundance of grass fuel loads between *C. intratropica* populations at Dukaladjarranj and a well-documented remnant population in Kakadu, growing on a fire-sheltered sand sheet adjacent to Round Jungle^25,52^ (Fig. S1). In July 2016, at each site we established 32 circular plots (10 m radius), each centred on a mature *C. intratropica* tree, selected to cover the spatial extent of each population^25,52^. In each plot, grass biomass was sampled from four 0.5 × 0.5 m quadrats that were placed 2, 4, 6 and 8 m from the central tree, in a spiral (one north, the next one east, the next one south and the last one west). Fuel weight was recorded using a spring balance, then converted to dry weight using site average water content determined by oven drying of subsamples.

### Dendrochronology at Dukaladjarranj

In 2015, dendrochronological samples were collected across 1700 m of the two transects at Dukaladjarranj (Fig. S4)*.* Cores were taken from 94 live trees ranging in diameter from 4 to 50 cm D_1.0_. We also took 54 disks or wedges from dead trees ranging from 6 to 30 cm diameter at breast height, sampling all dead trees that were safe to fell, and that were not rotten or badly fire damaged. The sample height above the ground was noted in all cases. The cores and stem sections were mounted and sanded using standard dendrochronological methods^79^. Digital images of samples were obtained using a 2400 DPI resolution flatbed scanner and analysed using the dendrochronology software CooRecorder and CDendro (Cybis Elektronik and Data, Saltsjöbaden, Sweden; http://www.cybis.se/forfun/dendro/). Where rings were ambiguous, samples were inspected under a binocular microscope in tandem with image analysis to ensure the accuracy of ring boundary placements. For living cores with missing inner rings, we used CooRecorder ‘pith estimator’ using the mean width of five preceding rings. Dead sections were cross-dated using CDendro and well-replicated chronologies^49^. As outlined below, establishment dates were estimated by adding the estimated number of years of growth required to reach the observed height using a height growth model developed using the juvenile growth data from Dukaladjarranj.

We calculated the annual height increment of tagged juveniles, measured annually over a period of three years (four measurement times) between 2006 and 2009 (see^30^ for details). There were 394 annual height growth increments from juveniles that were alive at both the start and finish of an interval. Initial height ranged from 2.4 to 189 cm (mean 29.5 cm). Mean annual height increments were 4.0, 5.3 and 6.2 cm for the first, second and third intervals, respectively. Linear modelling showed strong support for an influence of initial height on annual height increment (AIC for the model containing initial height was 51 lower than the intercept only model):

2 $${\text{Annual height increment}} = {2}.{57} + 0.{773}*{\text{initial height}}, {\text{r}}^{{2}} = 0.{125}$$

where height and annual height increment are in cm.

We used Eq. (2) to estimate the height–age relationship:

3 $${\text{Age}} = - {2}.{1} + {2}.0{24}*{\text{sqrt}}\left( {{\text{height}}} \right)$$

where age is in years.

Equation (3) was used to estimate the time required to attain the height at which sections or cores were sampled (the mean was 10 years for cores and 11 years for sections). The age was added to the dendrochronological age to estimate the establishment year.

### Research approvals

Experimental research and field studies on plants either cultivated or wild, including the collection of plant material, complied with relevant institutional, national, and international guidelines and legislation.

## Supplementary Information

Supplementary Figures.

## Data Availability

https://figshare.com/s/ff4c64dceb0acd6edec0 DOI identifier: 0.6084/m9.figshare.16921246.
