# Supplementary material for: Population collapse of a Gondwanan conifer follows the loss of Indigenous fire regimes in a northern Australian savanna
Source: Sci Rep. 2022 May 31;12:9081. doi: 10.1038/s41598-022-12946-3 (PMC9156674; doi:10.1038/s41598-022-12946-3)
Supplement: Supplementary file 1 — Supplementary Figures. [file 41598_2022_12946_MOESM1_ESM.docx]

**Supplementary Information**

**
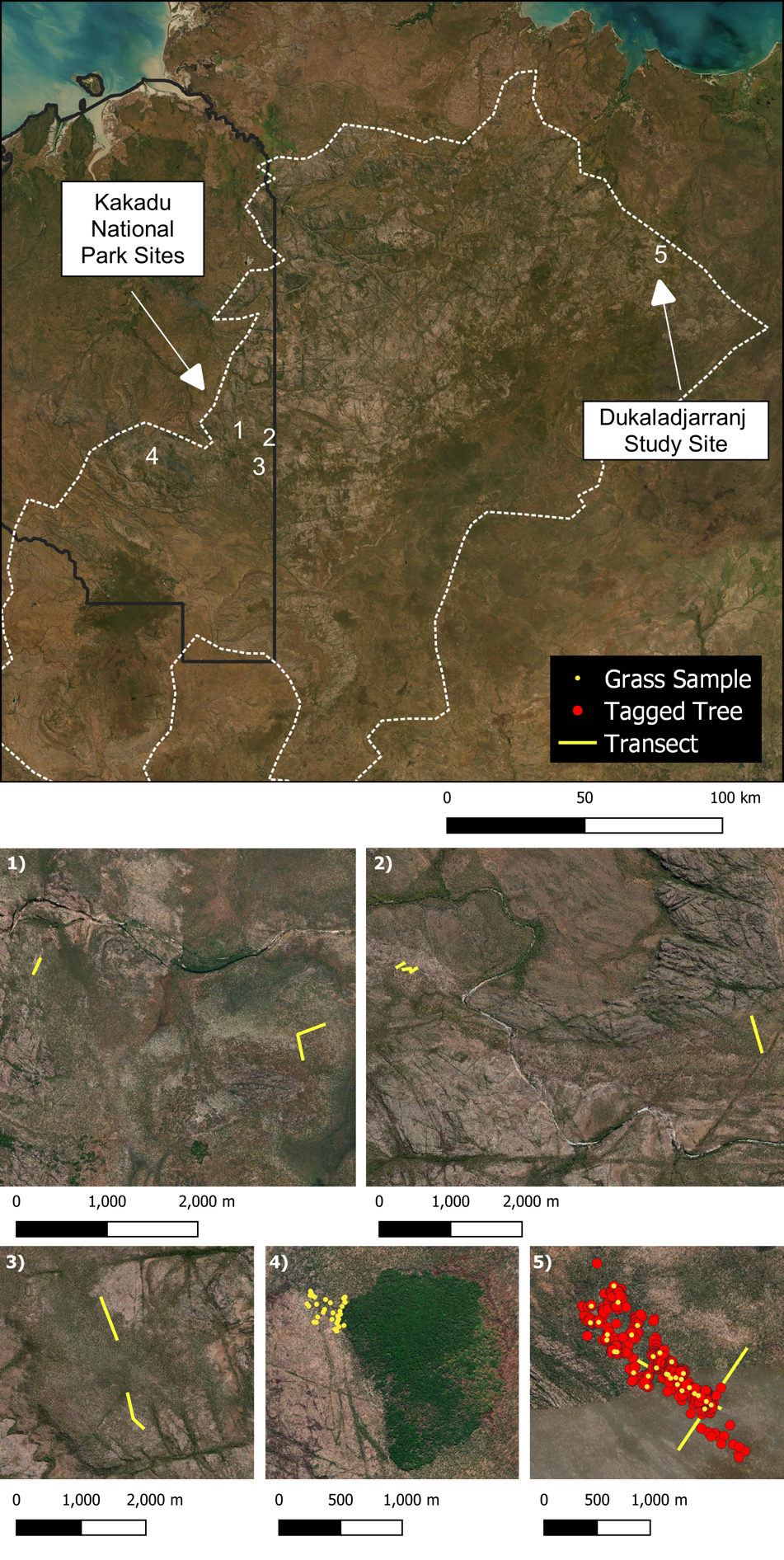
**

**Fig. S1.** Main panel. Location of transects (yellow lines) at three locations in Kakadu National Park (sites 1, 2 and 3) used to assess *C. intratropica* populations and grass fuel loads. Grass biomass was also assessed in circular plots surrounding living *C. intratropica* trees (yellow dots) at site 4 in Kakadu National Park. At the Dukaladjarranj Aboriginal estate (site 5) two crossing transects were used to assess *C. intratropica* population structures and grass fuel load sampling. Additionally, At Dukaladjarranj 1,092 *C. intratropica* individuals across all size classes, including juveniles, were tagged in mid-2006 to monitor growth and mortality and remeasured in mid-2019 (red circles). Grass biomass was assessed for a subset of these trees (yellow dots). In the main panel, the boundaries of Kakadu National Park (black line) and the Arnhem Plateau (white dashed line) are also indicated.


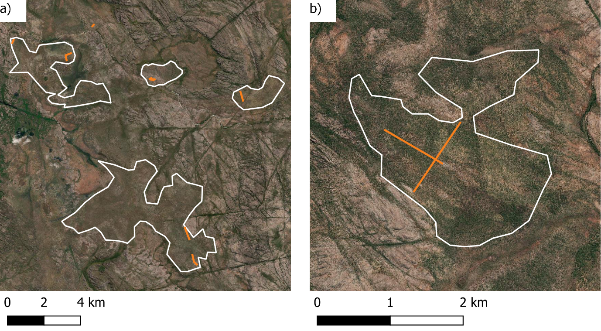


**Fig. S2.** Transects (orange) in *Eucalyptus* sand sheet savanna and associated polygons (white) selected for fire history analysis in (a) Kakadu National Park and (b) at Dukaladjarranj Aboriginal estate in central Arnhem Land.

**
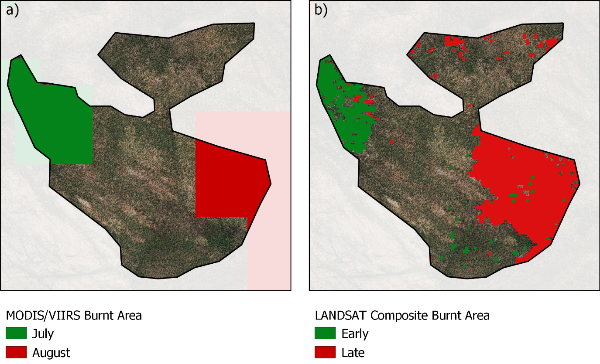
**

**Figure S3.** Example comparison of a) MODIS/VIIRS burnt area and b) LANDSAT composite burnt area for the site at Dukaladjarranj Aboriginal estate in central Arnhem Land for 2005.


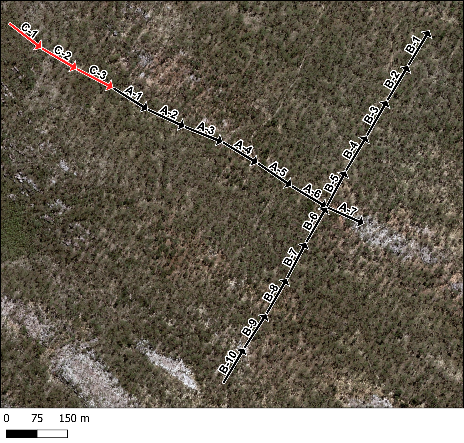


**Fig. S4.** Layout of the 100 m x 50 m segments comprising the two transects established to sample *Callitris intratropica* stand structures, grass fuel loads and dendrochronological survey at Dukaladjarranj Aboriginal estate in central Arnhem Land. Note three 100 segments (C1 to C3) in red) were not used for dendroecological sampling in 2015, nor the juvenile assessment in 2019.
